# Supplementary material for: Dynamic transcriptomic profiles of zebrafish gills in response to zinc supplementation
Source: BMC Genomics. 2010 Oct 11;11:553. doi: 10.1186/1471-2164-11-553 (PMC3091702; doi:10.1186/1471-2164-11-553)
Supplement: Additional file 2 — Interactive Direct Interaction Network representing the molecular interactions between zinc, copper, iron, calcium and proteins encoded by transcripts changed by zinc supplementation. Mini web-site containing index.html and hyperlinked pages in subdirectory describing a Direct Interaction Network automatically generated based on curated interactions contained within the proprietary PathwayArchitect database. Ovals represent proteins and the circles symbolize metal ions. Objects are coloured by their abundance in zebrafish at the time-point they were significantly different from the control is a scale from -4 fold (dark green) to +4 fold (dark red). Where significant differences were found at more than one time-point, the colour overlay shows expression at the first instance. Dark blue squares denote 'binding', and light blue squares 'expression'; green squares stand for 'regulation', green diamonds for 'metabolism', and green circles for 'promoter binding'. Arrow heads indicate directionality of the interaction where annotated. All nodes and edges can be further interrogated by selecting the relative area of the image. [file 1471-2164-11-553-S2.zip › PathwayArchitect Zn xs DIN/555853.html]

# BINDING:

|  |  |
| --- | --- |
| Type | BINDING |
| Effect | None |


---

|  |  |
| --- | --- |
| Score | 0 |


---

|  |  |
| --- | --- |
| Reference Count | 39 |


---

|  |  |
| --- | --- |
| Mechanism | Unknown |


---

|  |  |
| --- | --- |
| Reference:0 || Sentence | "Bovine calmodulin, bovine alpha-lactalbumin, and rabbit parvalbumin were found to bind specifically to 4, 1, and 2 Ca(2+) ions, respectively, in agreement with previously reported results obtained by using other physical methods." |
| PMID | 7847625 |
| Year | 1994 |
| Species | Human |
| Journal | Anal Chem |
| RefScore | 1 |
| Source | PArchNLP |
  |
|  |  |


---

|  |  |
| --- | --- |
 Reference:1 || Sentence | "Ca2+ and Mg2+ binding to rat parvalbumin was measured by means of the fluorescent Ca2+ indicator fluo-3 using a method developed earlier [Eberhard, M. & Erne, P. (1991) Eur. J. Biochem. 202, 1333-1338]." |
| PMID | 8200345 |
| Year | 1994 |
| Species | Rat |
| Journal | Eur J Biochem |
| RefScore | 1 |
| Source | PArchNLP |
  ||  |  |


---

|  |  |
| --- | --- |
 Reference:2 || Sentence | "At an ionic strength below 0.2 M, Kd values of Ca2+ binding to rat parvalbumin are approximately proportional to the ion concentration." |
| PMID | 8200345 |
| Year | 1994 |
| Species | Rat |
| Journal | Eur J Biochem |
| RefScore | 0 |
| Source | PArchNLP |
  ||  |  |


---

|  |  |
| --- | --- |
 Reference:3 || Sentence | "Kd values of Ca2+ binding were found to be about fourfold larger in the presence of Na+ as compared with K+, indicating that Na+ distinctly influences Ca2+ binding to rat parvalbumin." |
| PMID | 8200345 |
| Year | 1994 |
| Species | Rat |
| Journal | Eur J Biochem |
| RefScore | 1 |
| Source | PArchNLP |
  ||  |  |


---

|  |  |
| --- | --- |
 Reference:4 || Sentence | "Both Ca2+ and Mg2+ binding to parvalbumin are exothermic whereas Ca2+ and Mg2+ binding to fluo-3 are endothermic entropy-driven processes." |
| PMID | 8200345 |
| Year | 1994 |
| Species | Rat |
| Journal | Eur J Biochem |
| RefScore | 1 |
| Source | PArchNLP |
  ||  |  |


---

|  |  |
| --- | --- |
 Reference:5 || Sentence | "Parvalbumin binds two Ca2+, whereas many other members of the same superfamily bind four." |
| PMID | 3036821 |
| Year | 1987 |
| Species | Rat |
| Journal | J Biol Chem |
| RefScore | 0 |
| Source | PArchNLP |
  ||  |  |


---

|  |  |
| --- | --- |
 Reference:6 || Sentence | "Skin Ca2+-binding protein (SCaBP) was reported to be distinct from the Ca2+-binding parvalbumin (PV), however, more recently its amino acid sequence was shown to be identical to PV." |
| PMID | 3569514 |
| Year | 1987 |
| Species | Rat |
| Journal | FEBS Lett |
| RefScore | 1 |
| Source | PArchNLP |
  ||  |  |


---

|  |  |
| --- | --- |
 Reference:7 || Sentence | "There are two Ca(2+)-binding sites in parvalbumins, and there is some evidence for a third ion-binding site, adjacent to the CD site, in the rat species." |
| PMID | 8289291 |
| Year | 1994 |
| Species | Rat |
| Journal | J Mol Biol |
| RefScore | 1 |
| Source | PArchNLP |
  ||  |  |


---

|  |  |
| --- | --- |
 Reference:8 || Sentence | "In the case of the carp skeletal muscle, small molecular weight proteins including parvalbumin and two proteins of about 80K seemed to bind calcium ion." |
| PMID | 6715311 |
| Year | 1984 |
| Journal | J Biochem (Tokyo) |
| RefScore | 1 |
| Source | PArchNLP |
  ||  |  |


---

|  |  |
| --- | --- |
 Reference:9 || Sentence | "The effect of Ca2+ binding to parvalbumin was monitored by probes of conformation including absorption, fluorescence, circular dichroism (CD), infrared (IR) spectroscopy and differential scanning calorimetry." |
| PMID | 9154918 |
| Year | 1997 |
| Journal | Biochemistry |
| RefScore | 0 |
| Source | PArchNLP |
  ||  |  |


---

|  |  |
| --- | --- |
 Reference:10 || Sentence | "From these results we conclude that Ca2+ buffers with slow kinetics, such as PV, may cause biexponential decays in [Ca2+] transients, thereby complicating the analysis of endogenous Ca2+ binding ratios (kappaS) based on time constants." |
| PMID | 10835044 |
| Year | 2000 |
| Journal | J Physiol |
| RefScore | 2 |
| Source | PArchNLP |
  ||  |  |


---

|  |  |
| --- | --- |
 Reference:11 || Sentence | "Parvalbumin fragments from carp pI 4.47 parvalbumin corresponding to its residues 1--75 and 76--108 bind Ca2+ with affinities corresponding to Kd 0.9 . 10(-4) M and Kd 3 . 10(-3) M, respectively." |
| PMID | 623786 |
| Year | 1978 |
| Journal | Biochim Biophys Acta |
| RefScore | 1 |
| Source | PArchNLP |
  ||  |  |


---

|  |  |
| --- | --- |
 Reference:12 || Sentence | "The structure of pike parvalbumin pI 5.0, which binds Ca2+, and Mg2+ ions more tightly, is more stable than that of parvalbumin pI 4.2." |
| PMID | 6639084 |
| Year | 1983 |
| Journal | Arch Biochem Biophys |
| RefScore | 2 |
| Source | PArchNLP |
  ||  |  |


---

|  |  |
| --- | --- |
 Reference:13 || Sentence | "This work employed fluorescence energy transfer techniques to measure the distance between the Zn2+ (Cu2+) site and the stronger Ca(2+)-binding site in parvalbumin." |
| PMID | 1567990 |
| Year | 1992 |
| Journal | Biophys Chem |
| RefScore | 0 |
| Source | PArchNLP |
  ||  |  |


---

|  |  |
| --- | --- |
 Reference:14 || Sentence | "Significantly more labile heat was liberated than can be accounted for by the enthalpy change of Ca2+ binding to PA. 6." |
| PMID | 8246178 |
| Year | 1993 |
| Journal | J Physiol |
| RefScore | 1 |
| Source | PArchNLP |
  ||  |  |


---

|  |  |
| --- | --- |
 Reference:15 || Sentence | "Results of the molecular dynamics simulations indicate that all three modifications are disruptive to the integrity of the mutated Ca(2+) binding site in the whole parvalbumin protein." |
| PMID | 11297669 |
| Year | 2001 |
| Journal | Protein Eng |
| RefScore | 0 |
| Source | PArchNLP |
  ||  |  |


---

|  |  |
| --- | --- |
 Reference:16 || Sentence | "Despite the clear distinctions in the thermodynamic features, the conformational changes of toad parvalbumin are essentially the same as those of the two isotypes of bullfrog parvalbumins on Ca2+ binding and Mg2(+)-Ca2+ exchange." |
| PMID | 2156694 |
| Year | 1990 |
| Journal | Eur J Biochem |
| RefScore | 1 |
| Source | PArchNLP |
  ||  |  |


---

|  |  |
| --- | --- |
 Reference:17 || Sentence | "The Ca2+-binding parvalbumin (PV) is possibly involved in the relaxation of fast-twitch muscle fibers and believed to be a marker for early muscular disturbances." |
| PMID | 2960556 |
| Year | 1987 |
| Species | Rat |
| Journal | Exp Neurol |
| RefScore | 0 |
| Source | PArchNLP |
  ||  |  |


---

|  |  |
| --- | --- |
 Reference:18 || Sentence | "The observed shortening of half-relaxation time is due to PV and its ability to bind Ca2+, because a mutant protein lacking Ca(2+)-binding capacity did not promote any change in physiology." |
| PMID | 7604022 |
| Year | 1995 |
| Species | Rat |
| Journal | Proc Natl Acad Sci U S A |
| RefScore | 1 |
| Source | PArchNLP |
  ||  |  |


---

|  |  |
| --- | --- |
 Reference:19 || Sentence | "Comparison of the Ca2+-binding EF-hand domains of alpha-parvalbumin-Ca2+, calbindin-Ca2+, and calmodulin-Ca2+ shows that parvalbumin is probably the most rigid of the EF-hand proteins." |
| PMID | 9665701 |
| Year | 1998 |
| Species | Rat |
| Journal | Biochemistry |
| RefScore | 3 |
| Source | PArchNLP |
  ||  |  |


---

|  |  |
| --- | --- |
 Reference:20 || Sentence | "In addition, it has been shown that Ca2+ binding to parvalbumin parallels its activation of phosphodiesterase." |
| PMID | 195956 |
| Year | 1977 |
| Species | Rat |
| Journal | J Biol Chem |
| RefScore | 0 |
| Source | PArchNLP |
  ||  |  |


---

|  |  |
| --- | --- |
 Reference:21 || Sentence | "This result indicates that, despite the deletion of one Ca2+-binding domain, the remainder of the parvalbumin gene has maintained the structural pattern of the Ca2+-binding protein gene family." |
| PMID | 3009434 |
| Year | 1986 |
| Species | Rat |
| Journal | J Biol Chem |
| RefScore | 0 |
| Source | PArchNLP |
  ||  |  |


---

|  |  |
| --- | --- |
 Reference:22 || Sentence | "The AB-remodeled parvalbumins bind two Ca2+ ions with strong positive cooperativity (nH = 2) and moderate affinity ([Ca2+]0.5 = 2 microM), compared with [Ca2+]0.5 = 37 nM and nH = 1 for the wild-type protein." |
| PMID | 10491125 |
| Year | 1999 |
| Species | Rat |
| Journal | Eur J Biochem |
| RefScore | 1 |
| Source | PArchNLP |
  ||  |  |


---

|  |  |
| --- | --- |
 Reference:23 || Sentence | "An investigation of Ca2+-binding centers of parvalbumin II and III by analysing distant fine structure of X-ray absorption spectra of metal was performed." |
| PMID | 6472266 |
| Year | 1984 |
| Journal | Mol Biol (Mosk) |
| RefScore | 0 |
| Source | PArchNLP |
  ||  |  |


---

|  |  |
| --- | --- |
 Reference:24 || Sentence | "SR Ca2+ release was estimated from Delta[Ca2+] with a computational model that calculated Ca2+ binding to the major myoplasmic Ca2+ buffers (troponin, ATP and parvalbumin); buffer concentrations and reaction rate constants were adjusted to reflect fibre-type differences." |
| PMID | 12813151 |
| Year | 2003 |
| Species | Mouse |
| Journal | J Physiol |
| RefScore | 0 |
| Source | PArchNLP |
  ||  |  |


---

|  |  |
| --- | --- |
 Reference:25 || Sentence | "Ca2+ binding to PA will displace Mg2+ and hence the free myoplasmic concentration of Mg2+ ([Mg2+]i) will increase." |
| PMID | 7853224 |
| Year | 1994 |
| Species | Mouse |
| Journal | J Physiol |
| RefScore | 0 |
| Source | PArchNLP |
  ||  |  |


---

|  |  |
| --- | --- |
 Reference:26 || Sentence | "The experimental conditions were optimized with the recombinant parvalbumin test system before analyzing the Ca2+-binding properties of rat and murine parvalbumins in muscle tissue extracts." |
| PMID | 10036163 |
| Year | 1999 |
| Species | Mouse |
|  | Human |
|  | Rat |
| Journal | Anal Biochem |
| RefScore | 1 |
| Source | PArchNLP |
  ||  |  |


---

|  |  |
| --- | --- |
 Reference:27 || Sentence | "The Ca2+-binding parvalbumin has been purified for the first time from rat kidney." |
| PMID | 3519280 |
| Year | 1986 |
| Species | Rat |
| Journal | FEBS Lett |
| RefScore | 0 |
| Source | PArchNLP |
  ||  |  |


---

|  |  |
| --- | --- |
 Reference:28 || PMID | 15122922 |
| SourceID | 255122 |
| Species | Rat |
|  | Human |
| Experimental Condition | in-vitro |
| Description | The three dimensional solution structure of Human Alpha-Parvalbumin Refined with A Paramagnetism-Based Strategy was determined by using NMR spectroscopy. Parvalbumin Alpha was dissolved in a solution containing 1.5mM Parvalbumin U-15N,13C 100mM NaCl; 90% H2O, 10% D2 pH 7; For Cacapv; 0.6mM Parvalbumin U-15N; 100mM Na 90% H2O, 10% D2O; pH 7; For Cacapv; 1.5mM Parvalbumin U 15N,13C; 100mM NaCl; 90% H2 10% D2O; pH 7; For Cadypv; 0.6mM Parvalbumin U-15N; 10 NaCl; 90% H2O, 10% D2O; pH, 100 mM NaCl; 100 mM NaCl; 1 pH 7; For Cacapv; 0.6mM at 298K. The structure was determined by Distance Geometry, Simulate Annealing, Torsion Angle Dynamics, Pseudocontact Shifts, Residual Dipolar Coupling. 200 structures were calculated and 19 structures were submitted based on the structures with the Lowest Energy. PDB: 1RJV, MMDB: 27732. |
| Detection Method | three-dimensional-structure |
| Source | BIND |
  ||  |  |


---

|  |  |
| --- | --- |
 Reference:29 || PMID | 15122922 |
| SourceID | 255122 |
| Species | Rat |
|  | Human |
| Experimental Condition | in-vitro |
| Description | The three dimensional solution structure of Human Alpha-Parvalbumin (Minimized Average structure) was determined by using NMR spectroscopy. Parvalbumin Alpha was dissolved in a solution containing 1.5 mM Parvalbumin U-15N,13 100mM NaCl; 90% H2O, 10% D2 pH 7.0; For Cacapv; 0.6 mM Parvalbumin U-15N; 100mM Na 90% H2O, 10% D2O; pH 7.0; F Cacapv; 1.5 mM Parvalbumin 15N,13C; 100mM NaCl; 90% H2 10% D2O; pH 7.0; For Cadypv 0.6 mM Parvalbumin U-15N; 100mM NaCl; 90% H2O, 10% D2 pH 7.0; For Cadypv, 100 mM NaCl; 100 mM NaCl; 1 pH 7.0; For Cadypv at 298K. The NMR experiments conducted were 3D\_15N-Separated\_NOESY, 3D\_ 13C-Separated\_NOESY, 2D NOE 2D TOCSY, HNHA using field strength(s) of 700 MHz, 600 MHz, 500 MHz. The structure was determined by Distance Geometry; Simulate Annealing; Molecular Dynami Torsion Angle Dynamics Pseudocontact Shifts; Resid Diploar Couplings. 20 structures were calculated and 1 structures were submitted based on the structures with the Lowest Energy. PDB: 1RK9, MMDB: 27734. |
| Detection Method | three-dimensional-structure |
| Source | BIND |
  ||  |  |


---

|  |  |
| --- | --- |
 Reference:30 || PMID | 15122922 |
| SourceID | 255123 |
| Species | Rat |
|  | Human |
| Experimental Condition | in-vitro |
| Description | The three dimensional solution structure of Human Alpha-Parvalbumin Refined with A Paramagnetism-Based Strategy was determined by using NMR spectroscopy. Parvalbumin Alpha was dissolved in a solution containing 1.5mM Parvalbumin U-15N,13C 100mM NaCl; 90% H2O, 10% D2 pH 7; For Cacapv; 0.6mM Parvalbumin U-15N; 100mM Na 90% H2O, 10% D2O; pH 7; For Cacapv; 1.5mM Parvalbumin U 15N,13C; 100mM NaCl; 90% H2 10% D2O; pH 7; For Cadypv; 0.6mM Parvalbumin U-15N; 10 NaCl; 90% H2O, 10% D2O; pH, 100 mM NaCl; 100 mM NaCl; 1 pH 7; For Cacapv; 0.6mM at 298K. The structure was determined by Distance Geometry, Simulate Annealing, Torsion Angle Dynamics, Pseudocontact Shifts, Residual Dipolar Coupling. 200 structures were calculated and 19 structures were submitted based on the structures with the Lowest Energy. PDB: 1RJV, MMDB: 27732. |
| Detection Method | three-dimensional-structure |
| Source | BIND |
  ||  |  |


---

|  |  |
| --- | --- |
 Reference:31 || PMID | 15122922 |
| SourceID | 255123 |
| Species | Rat |
|  | Human |
| Experimental Condition | in-vitro |
| Description | The three dimensional solution structure of Human Alpha-Parvalbumin (Minimized Average structure) was determined by using NMR spectroscopy. Parvalbumin Alpha was dissolved in a solution containing 1.5 mM Parvalbumin U-15N,13 100mM NaCl; 90% H2O, 10% D2 pH 7.0; For Cacapv; 0.6 mM Parvalbumin U-15N; 100mM Na 90% H2O, 10% D2O; pH 7.0; F Cacapv; 1.5 mM Parvalbumin 15N,13C; 100mM NaCl; 90% H2 10% D2O; pH 7.0; For Cadypv 0.6 mM Parvalbumin U-15N; 100mM NaCl; 90% H2O, 10% D2 pH 7.0; For Cadypv, 100 mM NaCl; 100 mM NaCl; 1 pH 7.0; For Cadypv at 298K. The NMR experiments conducted were 3D\_15N-Separated\_NOESY, 3D\_ 13C-Separated\_NOESY, 2D NOE 2D TOCSY, HNHA using field strength(s) of 700 MHz, 600 MHz, 500 MHz. The structure was determined by Distance Geometry; Simulate Annealing; Molecular Dynami Torsion Angle Dynamics Pseudocontact Shifts; Resid Diploar Couplings. 20 structures were calculated and 1 structures were submitted based on the structures with the Lowest Energy. PDB: 1RK9, MMDB: 27734. |
| Detection Method | three-dimensional-structure |
| Source | BIND |
  ||  |  |


---

|  |  |
| --- | --- |
 Reference:32 || PMID | 11562941 |
| SourceID | 237547 |
| Species | Rat |
| Experimental Condition | in-vitro |
| Description | The three dimensional structure of Rat Parvalbumin Without the N-Terminal Domain was determined by crystallography. The crystal was grown by using Parvalbumin Alpha, Ammonium Sulfate 3m, Calcium Chloride 30mM pH 6.5, at 298K. X-ray data were collected using Rotating Anode as a radiation source and were 98.8% complete to a resolution limit of 1.440 angstroms and with an overall Rsym of 5.9%. This crystal contained 1 chain per asymmetric unit and belonged to the space group P 21 21 2. The structure was determined using Molecular Replacement and the modeled structure was refined to an R-factor = 16.9% and had an R-free = 20.0%. PDB: 1G33, MMDB: 17195. |
| Detection Method | three-dimensional-structure |
| Source | BIND |
  ||  |  |


---

|  |  |
| --- | --- |
 Reference:33 || PMID | 11562941 |
| SourceID | 237548 |
| Species | Rat |
| Experimental Condition | in-vitro |
| Description | The three dimensional structure of Rat Parvalbumin Without the N-Terminal Domain was determined by crystallography. The crystal was grown by using Parvalbumin Alpha, Ammonium Sulfate 3m, Calcium Chloride 30mM pH 6.5, at 298K. X-ray data were collected using Rotating Anode as a radiation source and were 98.8% complete to a resolution limit of 1.440 angstroms and with an overall Rsym of 5.9%. This crystal contained 1 chain per asymmetric unit and belonged to the space group P 21 21 2. The structure was determined using Molecular Replacement and the modeled structure was refined to an R-factor = 16.9% and had an R-free = 20.0%. PDB: 1G33, MMDB: 17195. |
| Detection Method | three-dimensional-structure |
| Source | BIND |
  ||  |  |


---

|  |  |
| --- | --- |
 Reference:34 || PMID | 15169955 |
| SourceID | 255158 |
| Species | Rat |
| Experimental Condition | in-vitro |
| Description | The three dimensional structure of Rat Alpha-Parvalbumin at 1.05 Resolution was determined by crystallography. The crystal was grown by using Parvalbumin Alpha, PEG, Ammonium Sulfate, Sodium Acetate pH 4.6, at 298K,. X-ray data were collected using synchrotron radiation and were 99.2% complete to a resolution limit of 1.050 angstroms with an overall Rsym of 6.0%. This crystal contained 3 chains per asymmetric unit and belonged to the space group P 21 21 21. The structure was determined using Molecular Replacement and the modeled structure was refined to an R-factor = 13.3% and had an R-free = 16.2%. PDB: 1RWY, MMDB: 27762. |
| Detection Method | three-dimensional-structure |
| Source | BIND |
  ||  |  |


---

|  |  |
| --- | --- |
 Reference:35 || PMID | 15169955 |
| SourceID | 255159 |
| Species | Rat |
| Experimental Condition | in-vitro |
| Description | The three dimensional structure of Rat Alpha-Parvalbumin at 1.05 Resolution was determined by crystallography. The crystal was grown by using Parvalbumin Alpha, PEG, Ammonium Sulfate, Sodium Acetate pH 4.6, at 298K,. X-ray data were collected using synchrotron radiation and were 99.2% complete to a resolution limit of 1.050 angstroms with an overall Rsym of 6.0%. This crystal contained 3 chains per asymmetric unit and belonged to the space group P 21 21 21. The structure was determined using Molecular Replacement and the modeled structure was refined to an R-factor = 13.3% and had an R-free = 16.2%. PDB: 1RWY, MMDB: 27762. |
| Detection Method | three-dimensional-structure |
| Source | BIND |
  ||  |  |


---

|  |  |
| --- | --- |
 Reference:36 || PMID | 15287728 |
| SourceID | 255159 |
| Species | Rat |
| Experimental Condition | in-vitro |
| Description | The three dimensional structure of Rat Alpha-Parvalbumin S55dE59D MUTANT was determined by crystallography. The crystal was grown by vapour diffusion using Parvalbumin Alpha, at 173K. X-ray data were collected using Rotating Anode as a radiation source and were 99.0% complete to a resolution limit of 2.000 angstroms and with an overall Rsym of 5.5%. This crystal contained 1 chain per asymmetric unit and belonged to the space group P 21 21 2. The structure was determined using Molecular Replacement and the modeled structure was refined to an R-factor = 18.1% and had an R-free = 22.1%. PDB: 1S3P, MMDB: 29340. |
| Detection Method | three-dimensional-structure |
| Source | BIND |
  ||  |  |


---

|  |  |
| --- | --- |
 Reference:37 || PMID | 15287728 |
| SourceID | 301843 |
| Species | Rat |
| Experimental Condition | in-vitro |
| Description | The three dimensional structure of Rat Alpha-Parvalbumin S55dE59D MUTANT was determined by crystallography. The crystal was grown by vapour diffusion using Parvalbumin Alpha, at 173K. X-ray data were collected using Rotating Anode as a radiation source and were 99.0% complete to a resolution limit of 2.000 angstroms and with an overall Rsym of 5.5%. This crystal contained 1 chain per asymmetric unit and belonged to the space group P 21 21 2. The structure was determined using Molecular Replacement and the modeled structure was refined to an R-factor = 18.1% and had an R-free = 22.1%. PDB: 1S3P, MMDB: 29340. |
| Detection Method | three-dimensional-structure |
| Source | BIND |
  ||  |  |


---

|  |  |
| --- | --- |
 Reference:38 || Sentence | Parvalbumin binds myoplasmic Ca(2+) during contractions, reducing calcium concentration and enhancing relaxation. |
| Year | 2006 |
| PMID | 16391345 |
| Journal | J Exp Biol |
| RefScore | 1 |
| Source | PArchNLP |
  |


---

|  |  |
| --- | --- |
